# Advances in Novel Therapeutic Strategies for Pulmonary Arterial Hypertension

**DOI:** 10.1155/carj/8046858

**Published:** 2026-04-08

**Authors:** Yalei Wang, Jiacheng Wu, Qian Ma, Hao Chen, Erha Lama, Yulu Yang, Jianwu Huang, Changhu Liu, Xiaofei Ni, Zhihua Qiu, Zihua Zhou

**Affiliations:** ^1^ Department of Cardiology, Union Hospital, Tongji Medical College, Huazhong University of Science and Technology, Wuhan, Hubei, China, hust.edu.cn; ^2^ Hubei Key Laboratory of Biological Targeted Therapy, Union Hospital, Tongji Medical College, Huazhong University of Science and Technology, Wuhan, Hubei, China, hust.edu.cn; ^3^ Hubei Provincial Engineering Research Center of Immunological Diagnosis and Therapy for Cardiovascular Diseases, Union Hospital, Tongji Medical College, Huazhong University of Science and Technology, Wuhan, Hubei, China, hust.edu.cn; ^4^ Key Laboratory of Biological Targeted Therapy, Huazhong University of Science and Technology, Ministry of Education, Wuhan, Hubei, China, meb.gov.tr; ^5^ Department of Cardiology, Renmin Hospital of Wuhan University, Wuhan, Hubei, China, rmhospital.com

**Keywords:** E-selectin, immunotherapy, molecular-targeted therapy, nanoparticles, pulmonary arterial hypertension, receptor NOTCH3

## Abstract

Pulmonary arterial hypertension (PAH) is a progressive, life‐threatening disease characterized by vascular remodeling, endothelial dysfunction, and chronic inflammation. Despite existing targeted therapies, long‐term outcomes remain poor, underscoring the need for more effective treatment. This review summarizes recent advancements in PAH therapy, emphasizing the integration of conventional medicine of the key pathways with nanoparticle‐mediated drug delivery systems (nano‐DDS), transforming growth factor beta/bone morphogenetic protein receptor type II, tyrosine kinase–based interventions, immunotherapies targeting inflammation and immune imbalance, and emerging molecular targets, including NOTCH3/HES‐5 and E‐selectin. Nano‐DDS has improved drug bioavailability and targeting, opening new therapeutic possibilities. This article also discusses innovative approaches, such as endothelial‐like progenitor cell–based cell therapy and the ETRQβ‐002 vaccine. Future advancements in PAH treatment may result from the integration of conventional drugs, nanotechnology, and immunotherapy through multidisciplinary efforts.

## 1. Introduction

Pulmonary hypertension (PH) is defined by a mean pulmonary arterial pressure (mPAP) > 20 mmHg at rest and represents a shared hemodynamic manifestation of diverse underlying conditions. It is clinically classified into five groups based on distinct pathophysiological mechanisms and etiologies, with specific diagnostic hemodynamic criteria outlined in Table 1. Among these, pulmonary arterial hypertension (PAH), designated as WHO Group 1, is characterized by the presence of precapillary PH in the absence of other major causative factors [1, 2]. PAH exhibits a higher incidence in females; however, males tend to have a worse prognosis [3]. Pulmonary vascular remodeling is the central pathophysiological hallmark of PAH, driven by endothelial cell (EC) dysfunction and the aberrant proliferation of pulmonary artery smooth muscle cells (PASMCs). ECs transition from an apoptotic to a hyperproliferative, antiapoptotic phenotype and acquire mesenchymal features through endothelial–mesenchymal transition (EndoMT), thereby driving vascular wall thickening and fibrosis during occlusive arteriolar remodeling. These dysfunctional ECs exhibit reduced nitric oxide (NO) production and increased endothelin‐1 (ET‐1) release. Through their interactions with immune and PASMCs, ECs critically promote disease progression and emerge as central drivers of PAH pathophysiology [4]. In severe cases, enlargement of the right heart may develop [5]. Several signaling pathways contribute to the development of PAH. ET‐1 is a strong vasoconstrictor and a growth factor. The ET‐A receptor, predominantly located on smooth muscle cells, facilitates vasoconstriction and cellular proliferation [6]. The phosphodiesterase‐5 (PDE5) pathway also plays a crucial role. In PAH, PDE5 is overactive, soluble guanylate cyclase (sGC) function is impaired, NO levels decrease, and oxidative stress increases. These alterations disrupt the vascular balance and lead to excessive blood vessel narrowing [7]. Endothelial dysfunction also lowers prostaglandin I2 synthase levels, thereby reducing the ability of blood vessels to dilate [8]. The Ras homolog family member A (RhoA)/Rho kinase (ROCK) signaling pathway is critical for regulating vascular smooth muscle contraction, endothelial function, and inflammatory responses [9].

**TABLE 1:** The hemodynamic diagnostic criteria of PH.

Categories	Hemodynamic characteristics	Explanation
PH	mPAP > 20 mmHg	The threshold for diagnosis

Precapillary PH	mPAP > 20 mmHg PVR > 2WU PAWP ≤ 15 mmHg	Pulmonary vascular disease–associated PH

Isolated postcapillary PH	mPAP > 20 mmHg PVR ≤ 2WU PAWP > 15 mmHg	Left heart disease–associated PH

Combined post‐ and precapillary PH	mPAP > 20 mmHg PVR > 2WU PAWP > 15 mmHg	Left heart disease with significant pulmonary vascular remodeling

Unclassified PH	mPAP > 20 mmHg PVR ≤ 2WU PAWP ≤ 15 mmHg	Typically due to increased pulmonary blood flow in conditions like congenital heart disease, liver disease, or hyperthyroidism, this form does not fall within the prior categories

Exercise PH	mPAP/CO slope > 3 mmHg·min·L^−1^ between rest and exercise	Assess abnormal pulmonary vascular responses during exercise

Abbreviations: CO, cardiac output; mPAP, mean pulmonary artery pressure; PAWP, pulmonary artery wedge pressure; PH, pulmonary hypertension; PVR, pulmonary vascular resistance.

Targeted therapies are now the standard for PAH, but the 5‐year survival rate remains between 50% and 86%, highlighting the need for better treatment [10]. Therefore, there is an urgent need to develop novel treatments. Current guidelines recommend drugs that target the key pathways. Endothelin receptor antagonists (ERAs) such as bosentan exhibit low bioavailability (∼50%) and significant hepatotoxicity. The newer ERA, macitentan, demonstrates reduced hepatotoxicity but retains teratogenic potential [11]. Phosphodiesterase‐5 inhibitors (PDE5i), including sildenafil (SDF) and tadalafil (TAD), rely on endogenous NO for their therapeutic effect, often necessitating higher doses to achieve optimal outcomes. These agents are associated with a relatively high incidence of systemic vasodilation–related adverse effects, including headaches, flushing, and nasal congestion. Prostacyclin drugs act quickly but wear off rapidly; stopping them suddenly can cause a dangerous increase in pulmonary pressure. Fasudil (FAD) is the only approved RhoA kinase inhibitor. In monocrotaline (MCT)‐PAH rats, FAD monotherapy outperforms bosentan and sildenafil, with no additional benefits from combination therapy [12]. FAD improves hemodynamics with minimal impact on blood pressure. Intravenous FAD reduces in‐hospital mortality in patients with right heart failure (OR = 0.258). However, the inhalational form has low lung deposition and limited efficacy, whereas the oral form suffers from inconsistent bioavailability and poor sustained release [13].

**FIGURE 1:**
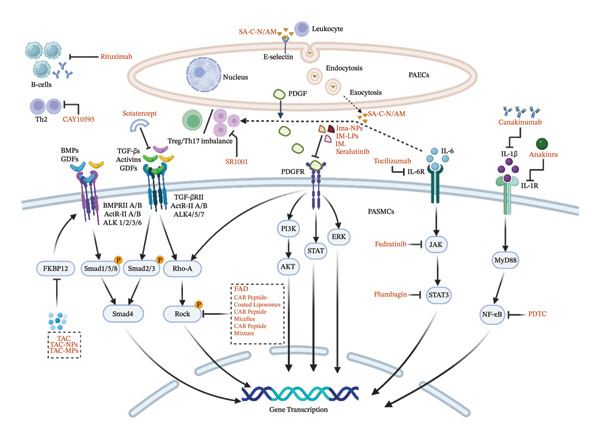
Therapeutic strategies targeting novel pathways. Above are PAECs and below are PASMCs. It presents the therapeutic strategies targeting emerging pathways in PAECs and PASMCs in PAH. PAECs: pulmonary artery endothelial cells; Sotatercept is a fusion protein that acts as a trap for activins and GDFs. Tacrolimus (TAC, or FK506) is a potent calcineurin inhibitor that binds to the intracellular protein FKBP‐12 and activates the BMPR2 pathway. TAC NPs: TAC was encapsulated into acetylated dextran nanoparticles (Ac‐Dex NP). TAC MPs: TAC was encapsulated into DPPC and DPPG; The tyrosine kinase inhibitor imatinib (IM) and seralutinib. IM‐LPs: IM‐loaded liposomes; Ima‐NPs: Imatinib‐loaded PLGA nanoparticles; fasudil (FAD) is a ROCK inhibitor. The RhoA/ROCK pathway can be activated either as a non‐SMAD pathway of TGF‐β or as a downstream effector pathway of PDGFR. Subsequently, FAD‐compliant CAR peptide–functionalized nanoparticles were introduced. Rituximab is a CD20‐targeting monoclonal antibody that depletes B cells. Inhibitors like SR1001 help restore balance of Treg/Th17. CAY10595 is an inhibitor of CRTH2, which could reduce Th2 activation and cytokine release and has therapeutic effects on PAH. PDTC: pyrrolidine dithiocarbamate; The NF‐κB inhibitor PDTC increases CD4+ Tregs, decreases CD8+ T and B cells, and restores immune balance. Tocilizumab is an IL‐6R antagonist. Ruxolitinib and fedratinib are JAK inhibitors. Plumbagin is a STAT3 inhibitor. Anakinra is an IL‐1 receptor antagonist. Canakinumab is an anti‐IL‐1β monoclonal antibody. In PAH, the expression of E‐selectin is upregulated in PAECs. E‐selectin promotes leukocyte adhesion and migration. SA‐C‐N/Am: nanoparticles coated with sialic acid (SA), a natural ligand for E‐selectin, and loaded with ambrisentan (Am). SA‐C‐N/Am are transcellularly transported by PAECs to PASMCs.

As research on PAH progresses, new therapies including immunomodulators, monoclonal antibodies, vaccines, cell therapy, and nanoparticle‐mediated drug delivery systems (nano‐DDS) have been used to overcome the limitations of conventional treatments in targeting, bioavailability, and sustained efficacy. Some have shown promising early results. This review provides a comprehensive overview of the therapeutic strategies targeting novel pathways, as shown in Figure 1, and presents new therapeutic strategies targeting classical signaling pathways, comparing their innovations and limitations, as shown in Tables 2 and 3, and provides a multidimensional reference for future research. It also discusses new targets such as NOTCH3/HES‐5 and E‐selectin, expanding potential PAH treatment options.

**TABLE 2:** Integration of conventional medicine with nanoparticle‐mediated drug delivery systems.

Targeted pathways	Therapeutic components	Nano‐DDS	Dosage	Route of administration	Animal model	Duration of treatment	Result	Innovations	Limitations	Ref.
ET‐1 pathway	Bosentan	PLGA	Loading 97.61%	Intratracheal	Wister Male Albino Rats	—	Sustained drug release with 12 h pulmonary vasodilation.	Minimize the first‐pass metabolism and improve systemic bioavailability.	Therapeutic efficacy in PAH models is unconfirmed, and long‐term data are lacking.	[14]

PDE5‐NO pathway	Sildenafil	PLGA and P(VS‐VA)‐g‐PLGA	100 μg	Intratracheal	U46619‐Rabbits	—	Significantly reduce PAP and PVRI.	Sustained‐release with no cumulative toxicity.	Unsuitable for acute exacerbations and low drug loading capacity.	[15]
		GlcA‐Lips	8 mg/kg	Intravenous	MCT‐PAH Rats	Every 48 h for 2 weeks	Reduce PVR and right ventricular hypertrophy, improving survival	GlcA‐Lips are pH‐sensitive liposomes with broad applicability.	Lack of multimodel validation and inadequate assessment of long‐term toxicity and immunogenicity.	[16]
		NanoMIL‐89	10 μg/mL	—	C57 Black Mice	—	Cause aortic dilation and suppress PASMC proliferation	Exhibits potential as an MRI agent and integrated diagnosis and therapy.	PAH model not used; lung targeting not validated.	[17]
	Tadalafil	PLGA	10 mg	Inhalation	In vitro	—	Sustained drug release over 24 h with deep lung deposition	Leucine incorporation enhances inhalation efficiency in nano‐micron structures.	The metabolism, toxicity, and efficacy of nanoparticles in the lungs are not yet fully investigated.	[18]

Prostacyclin pathway	Beraprost	PEG‐PLA/PLA	20 or 200 μg/kg	Intravenous	MCT‐PAH Rats and Hypoxia‐PAH Mice	21 days for MCT‐PAH Rats; 7 weeks for Hypoxia‐PAH Mice	Reduce PVR and right ventricular hypertrophy	Using the EPR effect for targeted delivery; the metal‐bridging strategy solves the encapsulation challenge	The preparation process is complex and hinders clinical translation	[19]
		PLGA	150 μg/kg	Inhalation	MCT‐PAH Rats and SuHx‐PAH Mice	2∼3 weeks for MCT‐PAH Rats; 2 weeks for SuHx‐PAH Mice	Reduce PVR and right ventricular hypertrophy, improving survival	Using microsprayer to enable intrapulmonary delivery; therapeutic effect lasting up to 2 weeks after single administration	Efficacy was not compared to existing drugs, and long‐term safety remains unevaluated	[20]
	Iloprost	LNPs	1, 2.5, 5 μM	—	Isolated mouse pulmonary artery	—	Dilates blood vessels with favorable safety profiles	Cationic LNPs improve encapsulation efficiency and cut dosage by half	Lack of in vivo data; LNPs may cause drug‐induced inflammation	[21]
	Treprostinil	LNPs	6 μg/kg	Inhalation	Hypoxic pulmonary artery constriction in Rats	—	Sustained dilation of the pulmonary artery	Prodrug‐loaded LNPs enable sustained release and have clinical potential	Lack of long‐term effectiveness and safety verification	[22]

*Note:* Nano‐DDS: nanoparticle‐mediated drug delivery systems; P(VS‐VA)‐g‐PLGA: poly(vinyl sulfonate‐co‐vinyl alcohol)‐graft‐poly(lactide‐co‐glycolide); MCT: monocrotaline; SuHx: sugen–hypoxia–normoxia; Lips: liposomes; EPR: the enhanced permeability and retention effect; LNPs: liposomal nanoparticles.

Abbreviations: mPAP, mean pulmonary artery pressure; MRI, magnetic resonance imaging; PAP, pulmonary artery pressure; PLGA, poly‐lactic/glycolic acid; PVR, pulmonary vascular remodeling; PVRI, pulmonary vascular resistance index; RVSP, right ventricular systolic pressure.

**TABLE 3:** Endothelial‐like progenitor cell (ELPC)–based cell therapy.

Cell‐type specificity	Dosage	Route of administration	Animal model	Duration of treatment	Result	Innovations	Limitations	Ref.
eNOS‐ELPCs	1 × 10^6^ cells for prevention group; 1.5 × 10^6^ cells for treatment group	Intravenous	MCT‐PAH Rats	3–5 weeks	Significantly reduces RVSP, improves survival, and sustains therapeutic efficacy	By integrating gene editing with cell therapy, it provides the first evidence that eNOS‐ELPCs can reverse PAH	eNOS gene expression lasted only 1 week, strategies are needed to overcome allogeneic cell rejection	[23]
	5 × 10^6^ cells	Intravenous	Hemodynamic PAH Rats	2 weeks	Significantly reduce PAP and reverse PVR	The hemodynamic PAH model closely reflects clinical pathology	Only single‐dose administration was studied; other regimens require further investigation	[24]

ELPCs‐nuRFP/CP	1.5 × 10^6^ cells	Intravenous	MCT‐PAH Rats	22–28 days	Cells > 80% remain in the lungs, reverse PVR, and improve survival	A COX1‐PGIS fusion protein was developed, increasing prostacyclin production twofold	Lack of validation with large animal models, the specific contribution ratios of prostacyclin and ELPCs remain unknown	[25]

BMPR2‐ELPCs	1 × 10^6^ cells	Intravenous	MCT‐PAH Rats	8–10 days	Reduce PVR and right ventricular hypertrophy	Achieve exosome‐mediated delivery of BMPR2	Cellular retention in the lungs lasted ≤ 6 h; long‐term efficacy and safety require further investigation	[26]

*Note:* eNOS‐ELPCs: eNOS‐transfected ELPCs; MCT: monocrotaline; ELPCs‐nuRFP/CP: ELPCs transfected with the COX1‐PGIS plasmid and the nuRFP lentiviral vector; BMPR2‐ELPCs: BMPR2‐overexpressing ELPCs.

Abbreviations: hemodynamic PAH, hemodynamic pulmonary arterial hypertension; PAP, pulmonary artery pressure; PVR, pulmonary vascular remodeling; RVSP, right ventricular systolic pressure.

## 2. New Therapeutic Strategies Targeting Classical Signaling Pathways

### 2.1. Targeting the ET‐1 Pathway

Researches on therapy targeting the ET‐1 pathway focus on three approaches: nano‐DDS, vaccines, and monoclonal antibodies. Combining nano‐DDS with bosentan enables inhaled delivery to the lungs and sustained release, improving bioavailability and reducing systemic toxicity. The ETRQβ‐002 vaccine and the humanized monoclonal antibody getagozumab show high specificity and long‐lasting effects, offering improved safety and convenience for treatment.

#### 2.1.1. PLGA‐NPs of Bosentan

Researchers prepared PLGA‐bosentan nanoparticles (NPs) using spontaneous emulsification solvent diffusion with dichloromethane/methanol (1:1), then formulated them into respirable controlled‐release polymeric colloids (RCRPC). NPs smaller than 500 nm escaped macrophage clearance. Nebulization generated droplets < 5 μm, achieving an alveolar deposition rate of 89.28%, which is much higher than standard solutions. In Wister male albino rats, intratracheal RCRPC showed a higher Cmax (1264.8 ± 323.68 vs. 105.1 ± 18.12 ng/mL, *p* < 0.0003) and similar Tmax (4.0 ± 1.41 h vs. 2.62 ± 1.49 h; *p* = 0.2296) compared to oral suspension. AUC_0_‐_t_ was 12.71 times higher (8649.8 ± 3319.7 ng·h/mL, *p* = 0.0030), indicating prolonged lung exposure. No lung damage was observed, confirming PLGA safety. Intratracheal delivery avoids first‐pass metabolism, reduces systemic effects, and improves bioavailability, while PLGA enables sustained release for less frequent dosing [14].

#### 2.1.2. ETRQβ‐002 Vaccine

To develop the ETRQβ‐002 vaccine, the second extracellular loop (ECL2) epitope peptide (ETR‐002) of the ET‐1A‐type receptor (ETAR) was conjugated with Qβ phage virus‐like particles (VLPs), alongside mAb A1C5. Immunization in animal models elicited robust antibody titers capable of effectively inhibiting ET‐1‐induced intracellular Ca^2+^ elevation, PASMC proliferation, and fibrotic mediator expression. This intervention resulted in reduced right ventricular systolic pressure (RVSP), improved pulmonary vascular remodeling, and attenuation of right ventricular hypertrophy with favorable safety profiles [27]. Long‐term efficacy evaluations verified that periodic booster immunizations maintained high antibody titers for up to 21 weeks. This vaccine has antiproliferative, anti‐inflammatory, and antifibrotic effects. It worked more effectively when administered before PAH onset; however, it maintained a wide therapeutic window. Its effect outperformed single ETAR blockers, although survival improvement was limited, necessitating further investigation of its underlying mechanism of action [28].

#### 2.1.3. Getagozumab

Getagozumab (GMA301) is the first humanized monoclonal antibody targeting ETAR (of the IgG4 type) that can specifically bind to human or monkey ETAR and inhibit Ca^2+^ influx induced by ET‐1. After a single intravenous dose in rhesus monkeys, getagozumab exhibited a prolonged half‐life of 9.9 days, over 10‐fold greater than that of conventional ERAs. It demonstrated favorable safety profiles, with no observed hepatotoxicity, edema, or hematological abnormalities. However, challenges remain in its clinical translation. At 15 mg/kg, the human dose is approximately 300 mg/patient, which increases cost concerns. Furthermore, repeated use may trigger antidrug antibodies, which require further evaluation [29].

### 2.2. Targeting the PDE5‐NO Pathway

Research on therapies targeting the PDE5‐NO pathway has advanced primarily along two fronts: nano‐DDS and cell therapy. Nano‐DDS extend beyond conventional nanoparticles by employing smart carriers such as glucose uronic acid–modified liposomes (GlcA‐Lips) and metal‐organic frameworks (MOFs), which enable targeted delivery to diseased cells, controlled drug release, and potential diagnostic functions. Meanwhile, engineered endothelial progenitor cells (EPCs) represent a novel cell therapy that repairs damaged blood vessels and continuously releases therapeutic factors, holding strong potential to reverse vascular remodeling.

#### 2.2.1. Sildenafil‐Nanoparticles (SDF‐NPs)

SDF‐NPs were prepared using a modified solvent evaporation technique with biodegradable polymers poly‐lactic/glycolic acid (PLGA) and charge‐modified poly(vinyl sulfonate‐co‐vinyl alcohol)‐graft‐poly(lactide‐co‐glycolide) (P(VS‐VA)‐g‐PLGA), forming submicron particles. In U46619‐induced acute PH rabbits, inhaled SDF‐NPs provided sustained drug release via electrostatic interactions, causing selective pulmonary vasodilation. The nanoparticles’ prolonged reduction of the PAP (pulmonary artery pressure) and PVRI (pulmonary vascular resistance index) lasted 150–180 min and ≥ 240 min, respectively, both longer than free SDF (90–120 min). SDF‐NPs have a slower onset than free SDF due to sustained drug release from the polymer, leading to delayed therapeutic effects and slower achievement of effective concentrations in the first 15–30 min. This is a key limitation of the formulation. The bronchoalveolar lavage fluid (BALF) analysis showed no acute toxicity or inflammation [15].

#### 2.2.2. TAD‐PLGA‐Nanoparticles (TAD‐PLGA‐NPs)

TAD‐PLGA‐NPs were prepared by emulsion solvent evaporation with 95.12% drug loading. The NPs were combined with lactose and mannitol to form inhalable powders having a mass median aerodynamic diameter (MMAD) of 1.4–2.8 μm, suitable for deep lung delivery. Drug release was sustained over 24 h and followed the zero‐order kinetic (*R* ^2^ = 0.98). Leucine modification improved aerosol performance by increasing fine particle fraction (FPF) and reducing particle cohesion. However, studies remain in vitro; in vivo evaluation of safety and efficacy is still needed [18].

#### 2.2.3. GlcA‐Modified Liposomes (GlcA‐Lips)

GlcA‐Lips were designed for intravenous delivery of SDF using thin‐film hydration and DSPE‐PEG conjugation with glucose uronic acid (GlcA) to target overexpressed glucose transporter‐1 (GLUT‐1) in pulmonary vascular. The inclusion of 6.9% pH‐sensitive DOPE enables rapid SDF release in acidic environments, offering two key benefits. First, increased glycolysis in remodeled pulmonary vessels acidifies the extracellular space, allowing pH‐responsive release that reduces premature drug leakage in circulation and boosts drug accumulation at the target site. Second, after uptake by PASMCs, liposomes enter endosomes and lysosomes, where lower pH triggers fast drug release for efficient intracellular delivery. In MCT‐induced PAH rats, GlcA‐Lips accumulated in PASMCs, as shown by confocal imaging. Compared to free SDF, GlcA‐Lips doubled the half‐life, mean residence time, and AUC, while halving systemic clearance. Treatment reduced PAP by 32.4%, decreased medial thickening by 41.3%, and improved survival. GlcA‐Lips suppressed the Warburg effect, increased eNOS and cGMP levels, and induced selective PASMC apoptosis. The platform shows promise for targeted PAH therapy, but long‐term toxicity and immunogenicity remain to be assessed [16].

#### 2.2.4. MOFs

NanoMIL‐89 was synthesized using a solvothermal method and loaded with SDF (1 mg/mL) in PBS for 16–18 h, yielding Sil@nanoMIL‐89 with a 17% drug loading capacity and over 90% efficiency. In isolated precontracted mouse aortae, free sildenafil induced rapid vasodilation within 60 min, whereas Sil@nanoMIL‐89 showed a 2–4‐h delay followed by sustained relaxation. Drug release was biphasic: 51% within 6 h and continued release from 72 to 96 h. Sil@nanoMIL‐89 was nontoxic to human blood outgrowth ECs (HBOECs) at up to 100 μg/mL, with no inflammation or cell damage. It can be modified with peptides for targeted therapy. The system remained stable under physiological conditions and, due to its low toxicity, biodegradability, and MRI compatibility, is promising for theranostic applications [17].

#### 2.2.5. eNOS‐Endothelial‐Like Progenitor Cells (eNOS‐ELPCs)

Transplantation of bone marrow–derived ELPCs prevents disease progression in MCT‐induced PAH rats. eNOS‐ELPCs can reverse PAH by reducing small pulmonary artery muscularization, promoting angiogenesis, restoring microvascular integrity, and improving survival [23]. A rat model of high pulmonary blood flow–associated PH, which is also called hemodynamic PAH, was developed using the right lung trileaflet resection to mimic congenital heart disease with a left‐to‐right shunt. Intravenous transplantation of eNOS‐enhanced EPCs enables targeted lung gene delivery, repairing endothelial dysfunction and activating the NO pathway, thereby providing dual therapeutic effects [24]. eNOS expression lasts only 1–2 weeks, and repeated high‐dose administration may prolong its effect. Allogeneic cell rejection remains a challenge in transplantation. The SAPPHIRE trial (NCT03001414), evaluating eNOS‐EPCs, was terminated early due to the COVID‐19 pandemic with only 12 participants enrolled. No significant difference was observed neither in the primary endpoint of a 6‐min walk distance (6MWD) (*p* = 0.19) nor in hemodynamic or cardiac structural parameters. However, 71.4% of patients receiving eNOS‐EPCs achieved a clinically meaningful improvement of ≥ 33 m in 6MWD, versus 40% in the placebo group. Improvement in the WHO functional class occurred only in the eNOS‐EPC group (43% vs. 0% in the placebo group). No treatment‐related serious adverse events were reported [30].

### 2.3. Targeting the Prostacyclin Pathway

Prostacyclin therapy has advanced through nano‐DDS, which prolong the pharmacological activity of key agents while improving target specificity and systemic stability. This overcomes key drawbacks of conventional treatment including short half‐life, frequent dosing, and high side‐effect risk.

#### 2.3.1. Beraprost‐Nanoparticles (BPS‐NPs)

Researchers prepared beraprost‐loaded nanoparticles (BPS‐NPs) using PLA and PEG‐PLA via solvent diffusion. The NPs targeted damaged pulmonary vessels through the enhanced permeability and retention (EPR) effect. In MCT‐induced PAH rats and hypoxia‐induced PAH mice, BPS‐NPs reduced pulmonary arterial remodeling and right ventricular hypertrophy. Weekly intravenous BPS‐NPs (20 μg/kg) achieved efficacy comparable to daily oral free beraprost (100 μg/kg). Both models showed significant improvements in pulmonary artery wall thickness and right ventricular hypertrophy (*p* < 0.05; *p* < 0.01). BPS‐NPs accumulated more in the lungs of MCT rats than free drug (*p* < 0.01), with retention lasting ≥ 5 days. FITC‐albumin lung accumulation increased 10‐fold (*p* < 0.001), confirming strong EPR effects in PAH. Reduced systemic distribution of BPS‐NPs may lower risks of side effects like headache and hypotension. No nanoparticle toxicity was reported in the study [19]. In another study, beraprost (BPS)‐loaded PLGA nanoparticles (BPS‐NPs) were prepared using the emulsion solvent diffusion method. The NPs also leveraged the EPR effect for selective delivery to injured pulmonary vasculature. Following intratracheal administration in MCT‐ or SuHx‐induced PAH rats, BPS‐NPs significantly reduced RVSP, attenuated right ventricular hypertrophy [RV/(LV + VS)], and decreased pulmonary arterial muscularization (*p* < 0.05). In MCT rats, BPS‐NPs markedly improved survival versus controls (65% vs. 27.8%). No significant toxicity was observed [20].

PLGA and PLA are highly hydrophobic, making them ideal for delivering hydrophobic drugs. PLGA degrades faster than PLA. Their hydrophobicity makes nanoparticles prone to recognition by the reticuloendothelial system (RES) and rapid clearance. PEGylation increases hydrophilicity, reduces RES uptake, and extends circulation time. This “stealth” effect enables longer blood circulation and enhanced the EPR effect [31].

#### 2.3.2. Iloprost‐Liposomal Nanoparticles (Iloprost‐LNPs)

Liposomes were prepared by the thin‐film rehydration method. Iloprost, negatively charged at physiological pH, was encapsulated into cationic liposomes (SA or DOTAP) via electrostatic interactions. Liposomal iloprost achieved the same vasodilatory effect as free iloprost at half the concentration (2.5 vs. 5 μM) in vitro vascular dilation assays. No significant cytotoxicity was observed in hPASMCs or A549 cells [21].

#### 2.3.3. TPD‐Lipid Nanoparticles (TPD‐LNPs)

Treprostinil was converted into an alkyl prodrug (TPD) via acid‐catalyzed esterification with alcohols of different chain lengths. TPD‐lipid nanoparticles (TPD‐LNPs) were formed by mixing TPD, squalene, DOPC, and cholesterol‐PEG2000 using a proprietary alcohol injection, followed by solvent diffusion. The LNPs enabled effective dispersion of poorly water‐soluble TPD into an inhalable solution. Cholesterol‐PEG2000 provided a “stealthy” coating, as mentioned above. After nebulization, TPD‐LNPs were delivered directly to the pulmonary vasculature, enabling dual sustained release through prodrug conversion and nanoparticle release. In hypoxic rats, inhaled TPD‐LNPs reduced plasma Cmax of treprostinil to about one‐tenth of free treprostinil, avoiding sharp drug peaks and reducing side effects like headache, nausea, and cough. PAP remained normal for up to 3 h [22].

#### 2.3.4. ELPCs‐nuRFP/CP

Rat bone marrow mononuclear cells were transfected with the nuRFP lentiviral vector for cell tracking and the COX1‐PGIS (CP) plasmid. After selection and induction, modified ELPCs (ELPCs‐nuRFP/CP) were obtained. Over 80% remained in the lungs after transplantation, delivering prostacyclin, reversing vascular remodeling, restoring the right ventricular neurotransmitter pathway, and improving survival. The COX1‐PGIS fusion protein doubled prostacyclin production, with one dose offering benefits for over 4 weeks [25].

## 3. Therapeutic Strategies Targeting Novel Pathways

### 3.1. Targeting the TGF‐β/BMPR2 Pathway

#### 3.1.1. The TGF‐β/BMPR2 Pathway in PAH

The TGF‐β superfamily regulates key cellular processes such as proliferation, differentiation, migration, and apoptosis. Its effects vary by the environment and cell type, with the latter being more influential [32–34]. As shown in Figure 1, this superfamily is divided into two main branches based on ligand and signaling specificity: TGF‐β/activin/nodal branch and BMP/growth differentiation factor (GDF) branch. Signaling occurs through type I (activin receptor‐like kinase, ALK4/5/7, ALK1/2/3/6) and type II (TGF‐βRII, ACTRII/B, BMPRII, AMHRII) serine/threonine kinase receptors. Ligands bind to type II receptor dimers, which then recruit and phosphorylate specific type I receptors, thereby forming an active heterotetrameric complex. The activated type I receptors phosphorylate receptor‐regulated Sma‐ and Mad‐related proteins (R‐SMADs): typically, SMAD1/5/8 in the BMP/GDF branch and SMAD2/3 in the TGF‐β/activin branch. The phosphorylated R‐SMADs form a complex with the common mediator SMAD4, translocate to the nucleus, and regulate target gene transcription. This process known as the canonical SMAD‐dependent pathway [35–37]. Non‐SMAD pathways also exist, including MAPK, PI3K‐Akt, and Rho‐GTPase signaling [38–40].

Endothelial‐to‐mesenchymal transition (EndoMT) is a key mechanism of vascular remodeling in PAH that is driven by an imbalance between the two branches [41]. Increased TGF signaling promotes EndoMT [42–44], whereas increased BMPR2 signaling has the opposite effect [45]. The TGF‐β superfamily also contributes to right ventricular remodeling in PAH, involving hypertrophy, fibrosis, and inflammation [46, 47]. In animal models, blocking TGF reduces right ventricular pressure and remodeling [48]. BMPR2 mutations are a major cause of hereditary PAH (HPAH) [37]. However, HPAH can also result from mutations in other TGF‐β/BMP pathway components, such as GDF2, activin A receptor like type 1 (ACVLR1), Endoglin (ENG), and SMAD9, which disrupting vascular homeostasis [49–52].

#### 3.1.2. Sotatercept

In patients with PAH, serum activin A levels are high and associated with higher mortality. Activin A signaling is active in SuHx mice and promotes PASMC proliferation in vitro. Monocrotaline pyrrole (MCTP) treatment increases ActRIIA expression, a receptor for activins and GDFs [53, 54]. Sotatercept (ActRIIA‐Fc) is a fusion protein that traps these ligands, blocks Smad2/3 activation, and inhibits abnormal cell growth, as shown in Figure 1. It also enhances BMP9 signaling and restores pathway balance. In preclinical studies, sotatercept significantly reduced mPAP, improved right ventricular hypertrophy, and inhibited vascular remodeling in MCT and SuHx rats, regardless of when it was administered [55]. Its anti‐inflammatory and antiproliferative effects are unaffected by BMPR2 mutations [56].

In the PULSAR trial, patients with PAH received Sotatercept (0.3 or 0.7 mg/kg every 3 weeks) or placebo. After 24 weeks, pulmonary vascular resistance (PVR) and six‐minute walk distance (6MWD) improved, and NT‐proBNP decreased [57]. Long‐term follow‐up confirmed sustained benefits and safety [58]. The Interim SOTERIA results also demonstrated consistent safety and efficacy over 2 years [59]. Post hoc analysis indicated no impact of BMPR2 status on the treatment response [60]. In STELLAR, Sotatercept lowered mPAP, reduced right heart load, improved pulmonary artery compliance, and enhanced exercise capacity. NT‐proBNP correlated strongly with mPAP and PVR and moderately with 6MWD [61]. It also reduced the risk of clinical worsening or death by 84% (HR = 0.16, *p* < 0.001) [62]. No serious immunogenicity‐related adverse events were reported, and the drug efficacy was unaffected [63]. In SPECTRA, Sotatercept increased peak VO_2_ by 102.74 mL/min, with lasting effects up to 48 weeks. The hemodynamic parameters and right ventricular structure improved. Common adverse events included epistaxis (12.3%), dizziness (10.4%), and telangiectasia (10.4%). Serious Treatment Emergent Adverse Events (TEAEs) occurred in 14.1% without treatment‐related deaths, indicating acceptable safety [64]. In HYPERION, early use of Sotatercept in PAH patients diagnosed within 1 year and in WHO functional classes II–III significantly reduced clinical worsening (hazard ratio 0.24) with rapid, robust effects when added to background therapy. This is the first evidence that Sotatercept provides meaningful clinical benefit early in PAH, expanding its therapeutic window [65]. In ZENITH, adding Sotatercept to PAH patients in high risk (WHO classes III–IV, REVEAL Lite 2 score ≥ 9) on maximum tolerated background therapy significantly reduced a composite endpoint of death, lung transplant, or hospitalization for PAH worsening ≥ 24 h (hazard ratio 0.24). This compelling efficacy led to early termination of the trial. These findings establish the clinical benefit of Sotatercept in the most severe spectrum of PAH, fulfilling a critical unmet need in advanced disease [66].

#### 3.1.3. Tacrolimus

Tacrolimus (TAC, or FK506) is a potent calcineurin inhibitor that binds to the intracellular protein FKBP‐12, blocks nuclear factor of activated T cell (NFAT) activation, and suppresses T‐cell‐mediated immunity by reducing cytokine release. Recent studies have indicated that TAC activates the BMPR2 pathway through dual mechanisms: Restoring inhibitor of differentiation 1 (ID1) and apelin expression, promoting vascular lumen formation, and improving endothelial function. Its efficacy has been verified in multiple PAH animal models [67]. Moreover, TAC improves right ventricular function in pulmonary artery banding (PAB) models by activating BMPR2 and inhibiting TGF‐β‐induced fibroblast proliferation and collagen production through ALK1, thereby reducing EndMT. These findings suggest that TAC may benefit not only PAH but also other conditions involving the right ventricular pressure overload, including Tetralogy of Fallot and group 2–4 PAH [68]. The treatments for PAH with TAC are shown in Figure 1. A small 16‐week trial (NCT01647945) demonstrated that low‐dose TAC (< 5 ng/mL blood concentration) exhibited good tolerability and partial restoration of BMPR2 signaling [69]. However, more extensive trials are required to evaluate the safety and efficacy comprehensively.

#### 3.1.4. TAC Nanoparticles (TAC NPs)

To improve pulmonary drug delivery, TAC was encapsulated into acetylated dextran nanoparticles (Ac‐Dex NP) via oil/water emulsion and solvent evaporation, yielding TAC‐loaded nanoparticles (TAC NPs). These were subsequently combined with mannitol to fabricate nanocomposite microparticles (nCmP) for dry powder aerosol administration. The TAC NPs exhibited a small particle size (∼200 nm) and a slightly negative zeta potential, facilitating enhanced penetration through the pulmonary mucus barrier. In vitro release studies showed that approximately 40% of TAC was released from the NPs over 12 h under physiological pH conditions, demonstrating sustained‐release behavior likely attributable to the pH‐sensitive degradation of Ac‐Dex. For safety evaluation, cytotoxicity was assessed in A549 cells. Free TAC showed no cytotoxicity at concentrations up to 10 μM, and TAC NPs exhibited comparable biocompatibility within the same concentration range [70].

#### 3.1.5. TAC Microparticles (TAC MPs)

TAC‐loaded dry powder microparticles (TAC MPs) were prepared using dipalmitoylphosphatidylcholine (DPPC) and dipalmitoylphosphatidylglycerol (DPPG) via spray drying. In A549 cells, TAC MPs were efficiently taken up within 24 h and localized to the nucleus. Dose–response analysis showed no cytotoxicity, supporting a favorable safety profile [71]. Compared with nCmP, this system offers better biocompatibility owing to its lung‐native components, prolonged lung retention, immune evasion, and structural stability. It has a simpler formulation and manufacturing process, enabling easier scale‐up for industrial production. It also achieves much higher drug loading (100 vs. 3.19 mg TAC/100 mg).

#### 3.1.6. BMPR2‐ELPCs

BMPR2‐overexpressing ELPCs (BMPR2‐ELPCs) were administered intravenously at 1 × 10^5^ cells per mouse on day 10 post‐MCT. Therapeutic effects were assessed 8–15 days later. Despite being cleared from the lungs within 24 h, BMPR2‐ELPCs significantly improved PAH, possibly through exosome release that sustains the signaling. This signifies the first successful delivery of a membrane‐bound receptor (BMPR2) using engineered cells, constituting a significant advancement in cell therapy. Future research should explore cryopreserved BMPR2‐enriched exosomes as a novel treatment strategy [26].

### 3.2. Targeting the Tyrosine Kinase Pathway

#### 3.2.1. Tyrosine Kinase Pathway in PAH

Platelet‐derived growth factor (PDGF) consists of four polypeptide chains that form five dimeric isoforms: PDGF‐AA,‐BB,‐CC,‐DD, and‐AB. These bind to PDGFR‐α or PDGFR‐β in homodimeric or heterodimeric complexes to activate downstream signaling [72, 73]. In cancer, PDGF signaling promotes remodeling of the tumor microenvironment and metastasis. In vascular diseases, it drives vascular smooth muscle cell (VSMC) migration, leading to plaque formation and pulmonary vascular remodeling. PDGFRs are receptor tyrosine kinases. PDGFR‐α is primarily expressed in mesenchymal cells, whereas PDGFR‐β is expressed in VSMCs and pericytes. Signaling outcomes vary by cell type. Activation of the pathway generates both stimulatory and inhibitory signals, with a net effect determined by their balance [74].

PDGF signaling interacts closely with the TGF‐β superfamily during PAH development. PDGF‐BB reduces BMPR2 expression through miR‐376b [75], whereas PDGF‐CC activates TGF‐β/Smad3 signaling, worsens TGF‐β pathway imbalance, and promotes disease progression [76]. The dual inhibition of PDGF and TGF‐β may offer a more effective treatment strategy. Hypoxia enhances PDGF signaling through HIF‐1α [77], while inflammation, estrogen, and lipopolysaccharide increase PDGFR expression [78]. The stromal cell–derived factor‐1 alpha (SDF‐1α)/C‐X‐C chemokine receptor type 4 (CXCR4) axis also interacts with PDGF signaling in PAH [79].

Aberrant PDGF signaling contributes directly to PASMC proliferation and migration, playing a crucial role in vascular remodeling [80]. C‐kit, a receptor tyrosine kinase, is expressed in c‐kit‐positive cells involved in vascular remodeling in idiopathic PAH (IPAH) [81]. The tyrosine kinase inhibitor imatinib (IM) targets PDGFR phosphorylation and inhibits c‐kit activity, reversing experimental PAH and improving remodeling in a dose‐dependent manner [82]. The treatment targeting tyrosine kinase pathways is shown in Figure 1.

#### 3.2.2. Imatinib

IM is a multitarget tyrosine kinase inhibitor (TKI) that inhibits BCR‐ABL, c‐kit, and PDGFR. It has transformed the treatment of chronic myeloid leukemia and gastrointestinal stromal tumors, ushering in an era of targeted therapy. Subsequent studies have revealed that IM also possesses the potential to treat PAH [83]. Preliminary studies have indicated that IM reverses experimental PAH by blocking PDGFR‐β phosphorylation [82]. Further studies suggest that it suppresses PASMC proliferation by inhibiting the PDGF‐Akt pathway [84]. IM also reduces vascular remodeling through the PDGFR‐β/TPH1/5‐HT and c‐kit pathways [85, 86] and may exert protective effects by modulating ET‐1 and NO signaling [87].

IMPRES research has validated IM as the first TKI for PAH. When added to standard therapies, IM improved exercise capacity and hemodynamics, with lasting effects [88]. It also enhanced right and left ventricular functions in advanced PAH [89]. Subgroup analysis suggested a greater benefit in patients with high PVR [90]. Combining IM with rapamycin reduced side effects and increased efficacy [91]. However, long‐term trials have revealed some major safety concerns. Most patients cannot tolerate IM due to adverse events, including subdural hematoma and death. Because of these risks, IM is not recommended for routine or off‐label use in PAH [92]. To improve safety, researchers have explored optimized dosing. A Phase II dose‐finding study indicated that 200 mg/day of oral IM was well tolerated. Total PVR took 40 days to return to baseline after treatment discontinuation, suggesting opportunities for better dosing strategies [93, 94]. Recently, an inhaled formulation of IM, AV‐101, has been developed. Preliminary findings indicate that it significantly lowers systemic exposure and improves tolerability compared with oral administration [95]. Currently, clinical trials are in progress [96]. Although anticoagulation is not routinely recommended in PAH management to prevent bleeding, its use requires careful monitoring (target INR ∼2.0) when employed, as demonstrated in studies of add‐on therapies like IM [97].

##### 3.2.2.1. Imatinib‐Loaded Liposomes (IM‐LPs)

IM‐loaded liposomes (IM‐LPs) were prepared using the transmembrane gradient method with spherical vesicles. Male Sprague‐Dawley rats received IM‐LPs via intratracheal instillation (10 mg/kg). IM‐LPs extended the half‐life threefold versus plain IM (12.68 ± 4.43 vs. 4.08 ± 1.01 h, *p* < 0.05) and prolonged MRT (10.38 ± 1.62 vs. 4.97 ± 0.72 h, *p* < 0.05). In vitro, plain IM released > 90% within 8 h; IM‐LPs showed sustained release (48.3 ± 0.97% over 48 h) and greater uptake by PASMCs. No significant cytotoxicity was observed [98].

##### 3.2.2.2. Imatinib‐Loaded PLGA Nanoparticles (Ima‐NPs)

Imatinib‐loaded PLGA nanoparticles (Ima‐NPs) were prepared by the emulsion solvent diffusion method. Ima‐NPs (1 mg/kg) were given via single intratracheal instillation to MCT‐induced PAH rats, significantly reducing RVSP and RV/(LV + S) (*p* < 0.05) and improving fully muscularized pulmonary arterioles (*p* < 0.05). In vitro, Ima‐NPs remained in PASMCs after extracellular drug removal, sustaining drug release and antiproliferative effects; free imatinib at the same dose was ineffective. A single low dose of Ima‐NPs achieved efficacy comparable to high‐dose imatinib (50 mg/kg). Ima‐NPs localized mainly in the pulmonary vascular wall, with little in liver or kidneys, and no significant cytotoxicity [99].

#### 3.2.3. Seralutinib

Seralutinib is a novel inhaled TKI that targets PDGFRα/β, CSF1R, and c‐kit. It demonstrates significantly greater potency than IM in inhibiting PASMC and fibroblast proliferation, with an IC50 up to 20 times lower. Seralutinib also exhibits higher selectivity for CSF1R and c‐kit, possibly because of structural modifications that enhance the ATP‐binding affinity. Unlike IM, it suppresses macrophage‐driven inflammation and upregulates BMPR2 by reducing miR‐135a‐5p and miR‐146a‐5p, thereby restoring TGF‐β/BMP signaling balance. Preclinical investigations using Su/Hx and monocrotaline pneumonectomy (MCTPN) rat models have validated their effectiveness [100].

The Phase II TORREY trial (NCT04456998) revealed that 24 weeks of seralutinib treatment significantly reduced PVR, mPAP, pulmonary arterial compliance (PAC), and cardiac workload, particularly in high‐risk patients and those with advanced functional classes. Safety data indicate good tolerability without fatal incidents and predominantly mild to moderate adverse effects [101]. The ongoing global Phase III PROSERA trial (NCT05934526) aims to further assess long‐term safety and efficacy. An imaging substudy demonstrated that seralutinib improves the distal‐to‐proximal pulmonary artery volume ratio, signifying a reversal of vascular remodeling and enhanced hemodynamics. These findings provide strong evidence of their therapeutic potential [102].

Administered by inhalation, seralutinib achieves lung concentrations 30 times higher than plasma levels, thus minimizing systemic exposure and side effects [100]. It does not impair endothelial barrier integrity and may be combined with other PAH therapies, including PDE5 inhibitors or ERAs, to enhance clinical outcomes [103]. Seralutinib is the first inhaled TKI specifically designed and in clinical development for the treatment of PAH.

### 3.3. Targeting Inflammatory Response and Immune System

#### 3.3.1. Targeting B Cells in PAH

B cells contribute significantly to vascular remodeling in PAH. CD20+ B cells constitute approximately 79.4% of the inflammatory infiltrates in vascular lesions and are concentrated around the affected vessels [104]. In MCT and hypoxia‐induced PAH models, B cells promote disease progression by producing pathogenic autoantibodies through bronchus‐associated lymphoid tissue [90]. Serum from patients with IPAH contains antivascular smooth muscle cell (anti‐VSMC) and anti‐EC antibodies [105, 106]. Patients with systemic sclerosis–associated PAH (SSc‐PAH) or connective tissue disease–associated PAH (CTD‐PAH) exhibit elevated levels of antiangiotensin II type 1 receptor and antiendothelin receptor A antibodies, which drive inflammation and vascular remodeling [107]. Peripheral blood CD19+ B cells from patients with IPAH exhibit elevated expression of genes associated with inflammation, immunological responses, and vascular remodeling [108]. Mast cells secrete interleukin IL‐6, which activates B cells and amplifies inflammation [109]. B cells also suppress regulatory T cells (Tregs), exacerbate autoimmune responses [110], and establish a positive feedback loop involving Bruton tyrosine kinase (BTK)–mediated activation, T follicular helper 17 (Tfh17) polarization, and further B cell stimulation, promoting immune imbalance and vascular remodeling [111]. In SSc‐PAH, B cells directly contribute to vascular pathology by secreting proangiogenic factors [112]. The treatment targeting B cells in PAH is shown in Figure 1.

##### 3.3.1.1. Rituximab

Rituximab is a CD20‐targeting monoclonal antibody that depletes B cells and has a favorable safety profile. It is extensively used in B‐cell lymphomas and autoimmune diseases. Case report has suggested its potential in treating lupus‐associated PAH (SLE‐PAH) [113]. The Phase 2 trial demonstrated that in SSc‐PAH, Rituximab improved the 6MWD particularly during B‐cell depletion and identified a subgroup of patients with low baseline levels of RF, IL‐2, and IL‐17 who exhibited more pronounced improvements in both 6MWD and PVR [114]. However, these findings require validation in larger studies owing to limitations such as small sample size and recruitment bias [115].

#### 3.3.2. Targeting T Cells in PAH

PAH is characterized by a T cell–mediated immunological imbalance, particularly the dysfunction of Tregs and elevation of T helper 17 cell (Th17) cells. CD4+ regulatory T cell (Tregs) facilitate the maintenance of immune balance and protect against PAH. Their exhaustion increases disease risk. Tregs secrete IL‐10, repair pulmonary artery ECs (PAECs), upregulate BMPR2, and inhibit inflammation and PASMC proliferation [116–118]. Loss of Treg function reduces autoimmune tolerance, promotes autoantibody production, activates effector T cells, and worsens vascular inflammation [119]. Impaired Treg responsiveness further limits their ability to suppress T cell and macrophage activation [120]. In PAH, Treg subpopulations are abnormal, with an increase in nonsuppressive Tregs (non‐Tregs). These cells lack immunosuppressive activity and instead generate proinflammatory cytokines, such as IL‐2 and IFN‐γ, promoting vascular inflammation and remodeling [121]. The higher prevalence of PAH in females may be associated with their greater dependence on Treg‐mediated protection. Tregs enhance COX‐2/PTGIS activity, increase PGI2 levels, and improve estrogen responsiveness, offering protective effects [122]. In the inflammatory environment of PAH, cytokines, including IL‐6, IL‐1β, and IL‐23, drive Treg‐to‐Th17 cell conversion, thereby accelerating disease progression [123].

The Treg/Th17 balance is disrupted in PAH, with elevated Th17 levels. Monocyte‐derived dendritic cells secrete IL‐23, promoting Th17 differentiation. IL‐17 further stimulates IL‐23 release, forming a feedback loop that sustains inflammation [124]. IL‐6 regulates this balance. Through the IL‐6/gp130/STAT3 pathway, it promotes Th17 development and inhibits TGF‐β‐induced Treg differentiation, worsening immune imbalance [125, 126]. The treatment targeting T cells in PAH is shown in Figure 1.

##### 3.3.2.1. Targeting Th17

Th17 cells drive chronic hypoxic PAH. Chronic hypoxia increases Th17 cell accumulation around pulmonary vessels by fourfold through the IL‐6/RORγt pathway. IL‐17A, produced by Th17 cells, directly stimulates PASMC migration and proliferation. Inhibitors such as SR1001 block Th17 differentiation and migration, demonstrating preventive and therapeutic potential in hypoxia‐induced PAH [127].

##### 3.3.2.2. Targeting Th2

In ovalbumin‐induced (OVA) PAH mice, circulating CD4+ Th2 cells increase and drive pulmonary artery myogenesis. The severity of myogenesis correlates with Th2 polarization, mediated by IL‐4 and IL‐13 [128]. Th2‐specific chemokine receptor CRTH2 (Chemoattractant Receptor Homologous Molecule Expressed on Th2 cells) is upregulated in PAH. CRTH2 knockout improves disease outcomes in preclinical mouse models. Similarly, treatment with the CRTH2 receptor antagonist CAY10595 reduces Th2 activation and cytokine release, demonstrating therapeutic benefits in established PAH [129].

##### 3.3.2.3. Targeting NF‐κB/IL‐6

NF‐κB promotes pulmonary vessel occlusion by inducing vascular cell apoptosis, inflammation, and immune dysregulation. The NF‐κB inhibitor, pyrrolidine dithiocarbamate (PDTC), reduces vessel occlusion, protects right heart function, and exhibits preventive and therapeutic effects in SuHx rat models. PDTC increases CD4+ Tregs, decreases CD8+ T and B cells, and restores immune balance. It is more effective than blocking downstream IL‐6 signaling and may serve as a promising target for severe obliterative PAH [130].

#### 3.3.3. Targeting JAK/STAT3/IL‐6 Pathway

IL‐6 binds to IL‐6Rα, triggering gp130 dimerization and activating Janus‐associated kinase (JAK) phosphorylation. This leads to STAT3 activation, which mediates EndMT, PASMC proliferation, inflammation, and vasoconstriction [131]. Elevated IL‐6 levels correlate with disease severity and reduced survival, even in mild PAH cases [132]. Similar associations were observed in children [133]. PASMCs serve as the primary source of IL‐6 in PAH. Recent studies have established a crosstalk between the JAK/STAT3 and TGF‐β/BMP pathways. In vitro data indicate that increased SERCA2a expression inhibits STAT3 phosphorylation, prevents its binding to the BMPR2 promoter, and enhances BMPR2 expression, restoring the signaling balance [134]. The gp130/STAT3 pathway also contributes to skeletal muscle atrophy in PAH. Stattic, a STAT3 inhibitor, effectively suppresses gp130‐induced myotube atrophy [135]. Prostacyclin drugs inhibit IL‐6 signaling by inducing suppressor of cytokine signaling 3 (SOCS3) expression [136]. Kynurenine metabolism promotes inflammation, vascular remodeling, and right heart dysfunction through the IL‐6/IL‐6Rα axis [137]. Therapies targeting the JAK/STAT3/IL‐6 pathway are shown in Figure 1.

##### 3.3.3.1. Tocilizumab

Tocilizumab is an IL‐6R antagonist used primarily for autoimmune diseases, including rheumatoid arthritis. Preclinical evidence supports its potential in PAH owing to its essential role in IL‐6 [138, 139]. Case reports indicated clinical and hemodynamic enhancements in patients with Adult‐Onset Still’s Disease‐Associated‐PAH (AOSD‐PAH) treated with tocilizumab [140]. However, a 6‐month trial revealed no significant advantage in idiopathic or hereditary PAH, although subgroup analysis suggested possible efficacy in CTD‐PAH [141]. Due to the small sample size and short duration, further trials are needed. Although Mendelian randomization did not indicate a causal role for IL‐6R variants in PAH risk, a clinical trial suggested potential benefit of tocilizumab in a small CTD‐PAH subgroup, underscoring the need for stratified approaches in future trials [142].

##### 3.3.3.2. Ruxolitinib

Current evidence links ruxolitinib, a JAK inhibitor, to an increased risk of PAH, independent of dosage [143, 144]. The mechanisms remain unclear but may involve immune dysregulation [145]. Certain studies have suggested that ruxolitinib interacts with PAH‐related proteins, including angiopoietin‐2 (ANGPT2), fibroblast growth factor 7 (FGF7), and 5′‐nucleotidase ecto (NT5E), through JAK/STAT3 or PI3K/Akt inhibition [146]. Given the limited available data, cardiopulmonary monitoring is recommended during treatment.

##### 3.3.3.3. JAK2 Inhibitor

Fedratinib TG101348 (Fedratinib), a selective JAK2 inhibitor approved for myelofibrosis, reduces hypoxia‐induced PASMC proliferation and vascular remodeling through the JAK2/STAT3/cyclin A2 pathway [147]. Magnolol, another JAK2 inhibitor, demonstrates comparable advantages in mitigating myocardial hypertrophy and fibrosis in hypoxic rats [148]. Additional research is required to validate their effectiveness in PAH.

##### 3.3.3.4. STAT3 Inhibitor

Plumbagin, a selective STAT3 inhibitor, reverses MCT‐ and SuHx‐induced PAH by targeting the STAT3/NFAT axis, upregulating BMPR2, inhibiting IL‐6 and ROCK1, modulating Src kinase activity, and exhibiting synergistic effects. It possesses a favorable safety profile and significant therapeutic potential, warranting further investigation [149].

#### 3.3.4. Targeting IL‐1β

Serum IL‐1β levels are elevated in patients with PAH and correlate with poor clinical outcomes [150]. In the MCT rats, IL‐1β acts as both an early inflammatory mediator and a chronic inflammatory driver. It promotes inflammation through the PI3K/Akt pathway, thereby establishing a positive feedback loop [151]. Pyroptotic PASMCs release IL‐1β and IL‐18, further stimulating their proliferation in a paracrine manner [152]. The NOD‐like receptor family pyrin‐domain containing 3 (NLRP3) inflammasome is the primary source of IL‐1β production in PAH [153]. Upon binding to IL‐1RI, IL‐1β activates the IL‐1RI/MyD88/NF‐κB signaling pathway, promoting PASMC proliferation through macrophage‐mediated inflammation [154, 155]. Caspase 8 activates the NLRP3 inflammasome, primarily in M1‐polarized macrophages rather than directly in PASMCs [156]. BMPR2 deficiency enhances IL‐1β/NF‐κB signaling, amplifying inflammatory responses [157]. Tregs can suppress IL‐1β, increase IL‐10, and block hPASMC cycle progression, thereby alleviating PAH [116]. However, IL‐1β has dual effects: It induces COX‐2 and increases prostacyclin (PGI2), but it also inhibits adenylate cyclase and reduces PGI2 signaling [158]. Therapies targeting the IL‐1β pathway are shown in Figure 1.

##### 3.3.4.1. Anakinra

Anakinra is an IL‐1 receptor antagonist initially used for autoimmune diseases, including juvenile idiopathic arthritis and familial Mediterranean fever [159], and demonstrates efficacy in type 2 diabetes [160]. Case reports have suggested its efficacy in AOSD‐PAH [161]. Preclinical findings indicate that anakinra reduces PASMC proliferation and macrophage‐driven inflammation in MCT and hypoxia mice, synergistically attenuating vascular remodeling [155]. A Phase I B/II trial evaluated subcutaneous anakinra (100 mg/day for 14 days) in seven patients with PAH and right heart failure. It ameliorated symptoms and inflammatory markers but exerted no significant effect on hemodynamics [162]. More extensive randomized trials are required because of study limitations.

##### 3.3.4.2. Canakinumab

Canakinumab is a long‐acting, humanized anti‐IL‐1β monoclonal antibody with high affinity and prolonged half‐life [163]. It blocks the interaction between IL‐1β and IL‐1RI by targeting a shared epitope [164]. Authorized for autoimmune diseases, canakinumab demonstrated cardiovascular benefits in the CANTOS trial by suppressing the IL‐1β/IL‐6/CRP cascade independent of lipid‐lowering therapy [165]. Although it remains preclinical in PAH, recent studies have indicated its potential. For instance, mice with hematopoietic DNMT3A deletion develop PAH with increased IL‐1β and macrophage activation. DNMT3A is the first gene identified to be significantly associated with APAH, a form of PAH that is often linked to inflammatory conditions such as scleroderma and that shows limited response to conventional vasodilator therapies. DNMT3A may represent a novel therapeutic target for this subtype [166]. Canakinumab significantly improved PAH in Dnmt3a^-^/^-^ mice, suggesting a promising strategy for APAH.

### 3.4. Targeting RhoA/ROCK Pathway

#### 3.4.1. RhoA/ROCK Pathway in PAH

The RhoA/ROCK signaling pathway contributes through two primary mechanisms: It enhances calcium sensitivity in PASMCs by inhibiting myosin light chain (MLC) phosphatase, leading to vasoconstriction, and promotes vascular remodeling through PASMC proliferation, endothelial dysfunction, and fibrosis. Multiple factors, including hypoxia, ET‐1, angiotensin II, 5‐HT, and PDGF, activate it. The RhoA/ROCK pathway suppresses eNOS, reducing NO production and worsening endothelial dysfunction. In patients with PAH, RhoA/ROCK activity in the lung tissue is twice that in healthy controls. Animal studies have indicated that ROCK activity increases with disease severity [9]. Emerging treatments targeting the RhoA/ROCK pathway are shown in Figure 1.

#### 3.4.2. Tissue‐Penetrating Homing Peptide‐CAR Peptide

CARSKNKDC (CAR) peptide is a cyclic, disulfide‐stabilized peptide that penetrates tissues and resists proteolysis. It binds to overexpressed heparan sulfate in pulmonary vessels, selectively targeting diseased vasculature without affecting normal vessels or other tissues [167]. Without covalent linkage, it enables a “bystander effect” when coadministered, enhancing drug delivery to target sites. CAR peptide binding may activate an endocytic transtissue transport pathway, named CendR‐pathway, boosting endothelial endocytosis and transvascular transport of drugs and macromolecules [168, 169]. In SU/Hx/Nx‐induced PAH rats, coadministration with FAD, imatinib, or SDF enhances pulmonary vasodilation with minimal impact on systemic blood pressure. CAR peptide alone shows no significant hemodynamic or toxic effects [170]. Due to its targeting ability, new CAR‐based therapies are under development.

##### 3.4.2.1. CAR Peptide–Coated Liposomes

Fasudil‐loaded liposomes were prepared by film hydration and active loading. They were modified with the CARSKNKDC (CAR) cyclic peptide, enabling targeted delivery to the pulmonary vasculature. Furthermore, the CAR peptide confers cell penetrating properties, promoting efficient internalization of the liposomes into PASMCs. In MCT‐induced PAH rats, CAR‐liposomes reduced mPAP by 40% for 6 h without affecting systemic blood pressure. In the SuHx rats, they showed sustained antihypertensive effects and higher targeting indices than controls. Half‐life was extended by 34‐fold, AUC increased by 16‐fold, and in vitro assays have demonstrated good specificity and safety for PASMCs [171]. Subsequently, CAR‐liposomes were further developed to coencapsulate FAD and superoxide dismutase (SOD), enabling dual targeting of the Rho kinase signaling and oxidative stress pathways. In vitro, drug uptake by PAECs and PASMCs nearly doubled [172]. In MCT‐ and SuHx‐induced PAH rat models, intratracheal CAR‐liposomes significantly reduced mPAP by 50% and 59.6%, alleviated right ventricular hypertrophy and vascular remodeling, and increased lung SOD levels 3.5‐fold, though sample sizes were small [173]. The limitations included no analysis of how liposomes cross the alveolar‐capillary barrier, no evaluation of CAR peptide immunogenicity or chronic toxicity, and a lack of data on the optimal peptide density and dosage.

##### 3.4.2.2. CAR Peptide Micelles

The same CAR peptide was used to modify DSPE‐PEG5000 micelles loaded with FAD (58% loading efficiency). Intratracheal delivery reduced mPAP by 60% without affecting systemic blood pressure. The half‐life of FAD was extended to approximately 7.93 h, which was about 15 times longer than that of ordinary FAD administered intravenously, suggesting its efficacy in MCT‐induced PAH rat models. Micelles were smaller (particle size 14 nm) than liposomes, resisted macrophage clearance, and penetrated lung tissue more effectively. Their glassy stiff core enabled sustained drug release; however, efficacy was only tested in MCT rats and drug loading was low [174].

##### 3.4.2.3. CAR Peptide Mixture

A lipid admixture containing FAD and DETA NONOate was prepared by the thin‐film hydration method and modified with the same CAR peptide (CAR‐DSPE‐PEG 2000). The CAR‐lipid mixture was administered intratracheally to SuHx rats every 48 h for 3 weeks. The results indicated that a combination treatment reduced mPAP and RVSP by ∼50%, improved heart function, and reduced pulmonary vascular remodeling and collagen buildup. No significant acute toxicity was observed [175].

#### 3.4.3. Starch‐Coated Magnetic Liposomes

Magnetic liposomes were prepared by solvent evaporation‐extrusion, coated with starch, and loaded with iron oxide particles and FAD, achieving 85% drug loading. In vitro, under a magnetic field (280 mT), magnetic liposomes rapidly migrated and accumulated, increasing PASMCs uptake threefold. Magnetic liposomes inhibited PASMC proliferation without cytotoxicity and released ∼80% of the drug over 5 days. In vivo, intratracheal delivery extended FAD’s half‐life 27‐fold and AUC 14‐fold. This was the first use of magnetic liposomes in PAH therapy, but the need for an external magnetic field limited its clinical use [176].

#### 3.4.4. Nanoerythrosomes (NERs)

NERs were prepared from red blood cells by removing hemoglobin through hypotonic lysis, forming pores, and loading FAD (20 mg/mL) before sealing. Nanoscale vesicles were generated by extrusion. NERs are taken up by PASMCs and PAECs, enabling targeted drug delivery. Made from natural red blood cell membranes, they show low macrophage uptake. In Sprague‐Dawley rats, NERs demonstrated good safety and biocompatibility. In vitro, FAD is released steadily over ∼48 h, with an initial burst of 11.3%. NERs inhibit Rho kinase by 53.4%, comparable to free FAD (65.6%). In vivo, after intratracheal administration, NERs extend FAD’s half‐life 6–8 times and increase AUC. Challenges include restricted red blood cell supply and elevated production expenses [177].

## 4. Emerging Therapeutic Targets in PAH

### 4.1. NOTCH3/HES‐5 Pathway

**FIGURE 2:**
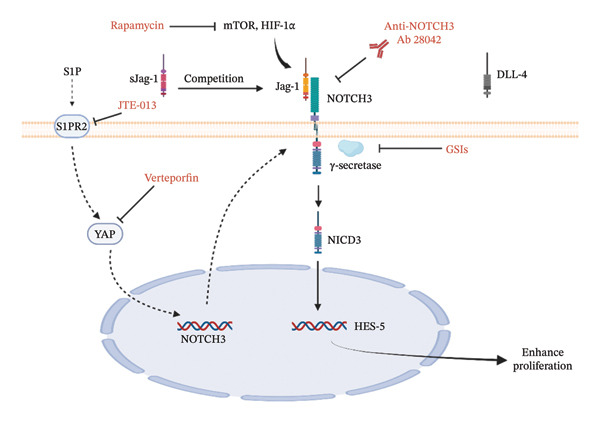
NOTCH3 pathway in PASMCs and therapeutic targeting of the NOTCH3 pathway. In PASMCs, Jag‐1 binds to the NOTCH3 receptor, triggering γ‐secretase to cleave NOTCH3 and release the intracellular domain NICD3. NICD3 then enters the nucleus and activates the gene HES‐5, which promotes PASMC proliferation. In contrast, DLL‐4 inhibits this process. Several strategies have been developed to inhibit NOTCH3 signaling: sJag‐1 blocks Jag‐1 binding; anti‐NOTCH3 Ab 28042 prevents Jag‐1 interaction; and γ‐secretase inhibitors (GSIs) stop NOTCH3 cleavage. In addition, other pathways such as S1P/S1PR2 and mTOR can be targeted using JTE‐013, verteporfin, or rapamycin to indirectly suppress NOTCH3 signaling. DLL‐4: Delta‐like ligand 4; sJag‐1: soluble Jag‐1.

NOTCH3 is a key biomarker in PAH, with upregulated signaling observed in PASMCs across multiple preclinical models from human IPAH. NOTCH3 upregulation correlates with disease severity [178, 179]. NOTCH3 is not merely involved in promoting PASMC proliferation; rather, a subset of VSMCs exhibiting high NOTCH3 expression has been identified as the cellular origin of obstructive vascular lesions in house dust mite extract (HDM) mouse model of PH. While this model differs from others, it provides a novel framework for investigating cellular heterogeneity in vascular remodeling, the relevance to human PAH warrants further investigation [180]. Genetic mutations in NOTCH3 have also been associated with an increased susceptibility to PAH in the pediatric population [181, 182]. As shown in Figure 2, Jagged‐1 (Jag‐1) and delta‐like ligand 4 (DLL‐4) are two primary ligands for NOTCH3. Jag‐1 activates NOTCH3 through an autocrine mechanism, facilitating its proteolytic cleavage and the subsequent release of NOTCH3 intracellular domain (NICD3). Hypoxia increases NOTCH3 levels through HIF‐1*α*, leading to NICD3 release and vascular remodeling [179]. This process causes transcriptional upregulation of the downstream target gene, HES‐5, thereby promoting PASMC proliferation. Conversely, DLL‐4 suppresses NOTCH3 cleavage by autocrine signaling, thereby inhibiting cell proliferation. Jag‐1 expression is elevated, while DLL‐4 expression is reduced in PASMCs in patients with PAH [183]. In HPAH, the NOTCH3/HES‐5 signaling axis contributes to vascular dysfunction by inducing endoplasmic reticulum (ER) stress and oxidative stress. Through ER stress mediation, this pathway enhances Ca^2+^‐dependent contraction, activates the ROCK pathway, and further exacerbates vascular remodeling [184]. NOTCH3 amplifies Ca^2+^ signaling through two distinct mechanisms: It can rapidly activate the transient receptor potential canonical 6 (TRPC6) channel through a noncanonical pathway or transcriptionally upregulate TRPC6 expression through the canonical pathway [185]. Moreover, NOTCH3 signaling interacts with other key molecular pathways, including the mTOR and S1P/S1PR2/YAP axis [186, 187].

Therapeutic strategies targeting NOTCH3 include upstream modulation using sJag‐1 to block Jag‐1 binding, thereby inhibiting PASMC proliferation and reversing dedifferentiation [188]. Direct targeting includes γ‐secretase inhibitors (GSIs) such as DAPT [178] and anti‐NOTCH3 Ab 28,042. The latter offers higher specificity and fewer gastrointestinal side effects [183]. Downstream approaches involve ROCK inhibitors (FAD), ER stress inhibitors (4‐PBA), and TRPC6 inhibitors. Future combination therapies may integrate NOTCH3 inhibitors with mTOR (Rapamycin), S1PR2 (JTE‐013), or YAP (Verteporfin) inhibitors for enhanced efficacy as shown in Figure 2.

### 4.2. E‐Selectin Pathway

E‐selectin is an adhesion molecule primarily located on vascular ECs and is minimally expressed under normal conditions. However, its expression rapidly increases when stimulated by inflammation, producing sE‐selectin, which enters circulation. sE‐selectin is a marker of endothelial activation [189]. E‐selectin promotes leukocyte adhesion and migration, thereby driving the inflammatory response. In PAH, E‐selectin levels rise through multiple signaling pathways. Its expression depends on BMPR‐II through the Smad pathway and is modulated by NF‐κB [190, 191]. In IPAH, overactivation of the migration inhibitory factor (MIF)/CD74 pathway in lung ECs boosts E‐selectin levels and maintains a chronic inflammatory state [192]. Under hypoxic conditions, E‐selectin levels increase in PAECs and trigger biliverdin reductase (BVR) translocation from the nucleus to the cytoplasm. This activates the E‐selectin/BVR pathway, stabilizing mitochondria, increasing Bcl‐2, and enhancing the BVR–BAX interaction, which inhibits BAX and prevents apoptosis. Consequently, PAECs proliferate abnormally, leading to vascular blockage [193]. Limited therapies directly target E‐selectin. Research indicates that MIF inhibitors, including isoxazole acetic acid methyl ester (ISO‐1) or anti‐CD74 antibodies, may be beneficial, with the latter exhibiting reduced cardiac toxicity [192]. Humanized anti‐E‐selectin monoclonal antibodies may represent a promising therapeutic strategy for future clinical applications.

#### 4.2.1. SA‐C‐N/Am

One study used chitosan nanoparticles coated with sialic acid (SA), a natural ligand for E‐selectin, and loaded them with ambrisentan (Am) to create SA‐C‐N/Am. Hypoxia upregulates E‐selectin on PAECs. SA targets nanoparticles to PAECs via E‐selectin binding, enabling transcellular transport to PASMCs, as shown in Figure 1. In hypoxic PAH lesions, nitroreductase (NTR) is overexpressed in a tumor‐like manner. The nitrophenyl group in the carrier is cleaved by NTR, triggering drug release in PASMCs. In hypoxia‐induced PAH rats, SA‐C‐N/Am colocalized with α‐SMA‐positive vessels. SA‐C‐N/Am showed prolonged half‐life (*t*_1_/_2_ = 1.72 h) and higher AUC_0_‐_24_h (39.11 mg·h/L) vs. control. It significantly reduced RVSP (*p* < 0.0001), RV/(LV + S) (*p* < 0.01), and improved TAPSE (*p* < 0.001), with near‐normal pulmonary arteriole muscularization. In vitro, SA enhanced nanoparticle uptake in HPAECs and transferred to cocultured HPASMCs. No cytotoxicity was observed [194]. SA‐C‐N/Am needs to further verify it in other preclinical models of PAH.

## 5. Summary and Future Perspectives

This article presents a comprehensive narrative review of emerging therapeutic strategies for PAH. PAH treatment is moving from vasodilation to a broader strategy targeting vascular proliferation, remodeling, and inflammation. Drug formulations have moved from small‐molecule drugs to nanoparticles, cell therapies, vaccines, monoclonal antibodies, and immunomodulators. Nano‐DDS increases pulmonary drug concentrations, reduces toxicity, and allows sustained release, which effectively addresses limitations of traditional chemical therapeutics, including short duration of action, low target specificity, and a high incidence of adverse effects. It represents a highly promising therapeutic approach [195, 196]. PLGA and PLA have high loading capacity for hydrophobic drugs due to their hydrophobic nature. PEGylation provides a “stealth” effect, helping NPs avoid immune clearance and stay in circulation longer. CAR peptide is a cyclic peptide that homes to diseased lung vessels and exerts a “bystander” effect. When coadministered with drugs, it improves targeting and efficacy without affecting systemic blood pressure. It can also be used to target NPs to the lungs. As shown in Table 2, integrating conventional drugs with nano‐DDS offers novel PAH treatment options. Nano‐DDS faces challenges including insufficient long‐term safety data, potential risks of pulmonary fibrosis and immune activation, lack of standardized manufacturing protocols, and relatively high production costs [197, 198]. As shown in Table 3, engineered ELPCs offer innovative methodologies for cell therapy. Transplanting engineered ELPCs in preclinical models repairs vascular endothelial injury and can prevent or reverse PAH. As shown in Figure 1, therapeutic strategies targeting novel pathways, including sotatercept, TAC, TKIs, rituximab, and anakinra, surpass conventional vasodilators and provide sustained therapeutic efficacy. Currently, several novel agents are undergoing clinical evaluation.

The combination of these innovative therapeutic strategies with nano‐DDS holds potential for improving treatment efficacy and warrants further investigation. For example, nano‐DDS may enhance the lung‐targeting capability of sotatercept, enabling dose reduction and lowering the overall drug development and treatment costs. New breakthroughs in PAH treatment necessitate the deeper integration of pharmacology, nanotechnology, and immunology to enhance basic research and clinical translation.

**Nomenclature**
PAH: Pulmonary arterial hypertension
TGF‐β: Transforming growth factor beta
BMPR2: Bone morphogenetic protein receptor type II
PH: Pulmonary hypertension
nano‐DDS: Nanoparticle‐mediated drug delivery systems
ELPCs: Endothelial‐like progenitor cells
mPAP: Mean pulmonary artery pressure
PASMCs: Pulmonary artery smooth muscle cells
ET‐1: Endothelin‐1
PDE5: Phosphodiesterase‐5
sGC: Soluble guanylate cyclase
NO: Nitric oxide
RhoA: The Ras homolog family member A
ROCK: Rho kinase
ERAs: Endothelin receptor antagonists
PDE5i: Phosphodiesterase‐5 inhibitors
SDF: Sildenafil
TAD: Tadalafil
FAD: Fasudil
MCT: Monocrotaline
EndoMT: Endothelial‐to‐mesenchymal transition
NPs: Nanoparticles
PLGA: Poly‐lactic/glycolic acid
MMAD: Mass median aerodynamic diameter
RCRPC: Respirable controlled‐release polymeric colloids
*C*_max_
: Maximum Plasma Concentration
*T*_max_
: Time to reach maximum plasma concentration
AUC_0‐t_
: Area under the plasma concentration–time curve from time zero to time *t*
P(VS‐VA)‐g‐PLGA: Poly(vinyl sulfonate‐co‐vinyl alcohol)‐graft‐poly (lactide‐co‐glycolide)
BALF: Bronchoalveolar lavage fluid
MOFs: Metal‐organic frameworks
DSPE‐PEG: 1,2‐Distearoyl‐sn‐glycero‐3‐phosphoethanolamine‐N‐[methoxy (polyethylene glycol)]
HPAH: High pulmonary blood flow–associated pulmonary hypertension
ELPCs: Endothelial‐like progenitor cells
EPCs: Endothelial progenitor cells
BPS: Beraprost
PLA: Poly(lactide)
SuHx: Sugen–hypoxia–normoxia
Lips: Liposomes
EPR: The enhanced permeability and retention effect
LNPs: Liposomal nanoparticles
MRI: Magnetic resonance imaging
RVSP: Right ventricular systolic pressure
DOTAP: 1,2‐di‐(9Z‐octadecenoyl)‐3‐trimethylammonium‐propane
ID1: Differentiation 1
OVA: Ovalbumin
PDTC: Pyrrolidine dithiocarbamate
SA: Stearic acid
TRE: Treprostinil
PVR: Pulmonary vascular resistance
6MWD: Six‐minute walk distance
HPAH: Hereditary pulmonary arterial hypertension
GDF: Growth differentiation factor
ALK: Activin receptor–like kinase
ENG: Endoglin
SMAD: Sma‐ and mad‐related proteins
TEAEs: Treatment emergent adverse events
TAC: Tacrolimus
PAB: Pulmonary artery banding
NFAT: Nuclear factor of activated T cells
Th17: T helper 17 cell cells
Tregs: Regulatory T cell
PAECs: Pulmonary artery endothelial cells
SOCS3: Suppressor of cytokine signaling 3
AOSD‐PAH: Adult‐onset still’s disease–associated‐PAH
CTD‐PAH: Connective tissue disease–associated‐PAH
ANGPT2: Angiopoietin‐2
FGF7: Fibroblast growth factor 7
NT5E: 5′‐nucleotidase ecto
NLRP3: NOD‐like receptor family pyrin domain containing 3
MLC: Myosin light chain
SOD: Superoxide dismutase
DN: DETA NONOate
HDM: House dust mite
Jag‐1: Jagged‐1
DLL‐4: Delta‐like ligand 4
NICD3: NOTCH3 intracellular domain
ER: Endoplasmic reticulum
TRPC6: Transient receptor potential canonical 6
DAPT: N‐[N‐(3,5‐Difluorophenacetyl)‐L‐alanyl]‐S‐phenylglycine t‐Butyl Ester
MIF: Migration inhibitory factor
BVR: Biliverdin reductase
Bcl‐2: B‐cell lymphoma 2
BAX: Bcl‐2‐associated X protein
ISO‐1: Isoxazole acetic acid methyl ester

## Author Contributions

Yalei Wang: writing–original draft, data curation, and investigation; Jiacheng Wu: methodology and visualization; Qian Ma: software and supervision; Hao Chen: investigation; Erha Lama: data curation; Yulu Yang: software and visualization; Jianwu Huang: conceptualization and validation; Changhu Liu: data curation; Xiaofei Ni: methodology; Zhihua Qiu: writing–review and editing, and validation; Zihua Zhou: writing–review and editing, funding acquisition, and conceptualization.

## Funding

This study was supported by the National Natural Science Foundation of China (Nos. 82070378, 82370352, and 82270534).

## Ethics Statement

The authors have nothing to report.

## Consent

The authors have nothing to report.

## Conflicts of Interest

The authors declare no conflicts of interest.

## Data Availability

No data were used for the research described in the article.
